# The role of university entrepreneurship support and student diversity in student entrepreneurial intention: a social identity theory perspective

**DOI:** 10.1371/journal.pone.0321651

**Published:** 2025-04-23

**Authors:** Tamara Ratkovic, Ivan Paunovic, Helena Šlogar

**Affiliations:** 1 Singidunum University, Faculty of Physical Education and Sports Management, Belgrade, Serbia; 2 Bonn-Rhein-Sieg University of Applied Sciences, Campus Rheinbach, Centre for Entrepreneurship, Innovation and SMEs (CENTIM), Rheinbach, Germany; 3 Singidunum University, Faculty of Health and Business Studies, Valjevo, Serbia; 4 University of Rijeka, Faculty of Economics and Business, Rijeka, Croatia; Instituto Politecnico Nacional, MEXICO

## Abstract

One of the most significant shortcomings of the extant literature is the dearth of conceptualizations and empirical testing pertaining to entrepreneurial diversity and the underlying social identities. Accordingly, this research aims to empirically investigate the influence of entrepreneurial support at the university level on students’ entrepreneurial intention from a diversity and social identity perspective. The research utilizes fsQCA (fuzzy set Qualitative Content Analysis) and a set of observable and underlying diversity factors (gender, age, previous academic performance, and parental educational attainment) to elucidate the causal structures that influence the formation of entrepreneurial intention among university students. The findings illustrate the multifaceted nature of the entrepreneurial intention formation, with first-generation status being a necessary condition for low entrepreneurial intention. The findings underscore the importance of university entrepreneurial support in the formation of entrepreneurial intention especially for younger students and first-generation students. The study contributes to the literature on entrepreneurial diversity and social identities in entrepreneurship by identifying the underlying factors that contribute to high and low entrepreneurial intention among university students.

## 1. Introduction

Recent findings from the extant literature reveal a considerable degree of heterogeneity in the factors and motivations underlying entrepreneurship across different social groups [1]. This heterogeneity is particularly evident among “untypical” entrepreneurs- the ones lacking self-efficacy and/or entrepreneurial role models. This highlights the need for greater attention in the literature on the relation between diversity and entrepreneurship. Diversity, encompassing factors such as gender, age, education, experience, nationality, culture, and others, as well as their interdependence within the socio-economic environment, plays a pivotal role in shaping the overall entrepreneurial ecosystem [2]. However, in entrepreneurship research, the diversity aspects and their interconnectedness are still poorly defined and represent a significant research gap in entrepreneurship research [3]. The present study is grounded in social identity theory, as initially defined by Tajfel [4] and subsequently developed within the framework of diversity management [5,6], wherein diversity components are categorized as observable and underlying. Building upon the identified research gaps in the entrepreneurship literature and drawing upon theoretical advancements in the domain of social identity and diversity, the present study aims to address the following research questions:

RQ1: What are the necessary conditions for high/low entrepreneurial intention among university students in terms of entrepreneurial support by university as well as observable and underlying students’ diversity factors?

RQ2: What equifinal causal structures explain the impact of entrepreneurial support by university and observable and non-observable students’ diversity factors on high/low entrepreneurial intention of university students?

A review of recent literature on the subject of entrepreneurial education reveals a critical perspective on the efficacy of such programs. For instance, the findings of a meta-analysis, indicate a lack of evidence supporting the claim that entrepreneurial education increases entrepreneurial intention or self-efficacy [7]. Conversely, the beneficial influence of entrepreneurial education on female students has been extensively substantiated in the academic literature [8,9]. However, there is a notable absence of research examining entrepreneurial intention in the university context from a diversity perspective. This is evidenced by the paucity of diversity variables in extant research on entrepreneurial intention in the university context, which has been predominantly focused on gender [10,11]. There is a need for further investigation into other observable (age, gender) and non-observable diversity factors (previous academic achievement, parental education level). In light of the significance of both diversity management and entrepreneurial education within the third mission of universities, this research gap merits greater attention, both empirically and conceptually. While previous entrepreneurship literature has provided a robust conceptualization of diversity aspects [3,12], there is a notable absence of empirical evidence examining the impact of entrepreneurial education on entrepreneurial intention from a social identity and diversity perspective. This represents a significant research gap.

The future of entrepreneurship will be characterized by greater diversity, including a narrowing of the gender gap in favor of increased female participation, as well as greater inclusivity with regard to age, socio-economic status (including migrant and refugee status), and ethnicity [13]. The authors further develop this line of thought, proposing that by 2030, entrepreneurship will become the primary economic vehicle for marginalized populations to survive, and that entrepreneurship education will support a more diverse array of marginalized populations. A review of the literature on entrepreneurial education reveals a dearth of research on diversity in this field. Despite numerous calls for greater diversity in entrepreneurship, both in terms of conceptual and social aspects [14] as well as spatial and population-related considerations [2], there has been no specific focus on diversity in entrepreneurial education, representing a significant research gap.

The article commences with an examination of the extant literature on social identity and diversity in entrepreneurship, entrepreneurial ecosystems and entrepreneurial education. It then reviews the literature on entrepreneurial intention and diversity in the university context as a specific case of entrepreneurial intention. The article then proceeds to delineate the research framework by introducing the diversity management literature, including the classification of diversity attributes to observable and underlying and how these factors relate to social identity theory. The subsequent section details the research sample, along with batteries of questions and a data calibration procedure in fsQCA software. The article then presents the results of the necessity and sufficiency analyses followed by a discussion of the theoretical and managerial contributions as well as potential avenues for future research and the limitations of the present study. Finally, the article offers concluding and closing remarks.

## 2. Literature review

### 2.1 Social identity and social identity theory

Social identity is defined as a component of an individual's self-concept, derived from their awareness of their affiliation with a particular social group, accompanied by the values and emotional significance attributed to it [4]. Notable group classifications, such as gender, profession, nationality, age, and religious affiliation [15,16], are included in the concept of social identity. Additionally, other stable attributes, including race, ethnicity, and veteran status [17] can also be incorporated. In the context of social identity research and theorizing, demographic attributes have been identified as the strongest predictors of group formation within organizations [15]. Social identity theory posits that social group memberships influence individuals’ values, emotions, and self-esteem [18]. In the present research, we use the social identity theory to explicate entrepreneurial intention of university students. By the adolescence, most young people have already established social identities, while continuing to work on personal and vocational identities [19].

The extant literature on social identities in the field of entrepreneurship is inconclusive as to what exactly constitutes a social identity, thus creating a significant a research gap. This research gap is based in the inconclusiveness of the general social identity theory regarding emerging and situated vs. pre-determined categories [15]. Recent research on social identities in entrepreneurship has identified and built upon the notion of emerging and situated social identity [20,21]. However, other relevant and important approaches that define social identity in entrepreneurship as the identification of prospective entrepreneurs with a specific pre-determined group, such as peers in academia [22]. Our approach to social identity is informed by this latter perspective, aligning with the classical sociological notion that social identity is a pre-defined classification of which an individual has explicit knowledge. However, the present research also goes beyond single category to examine the dynamic interplay of multiple social identities. This approach is informed by the traditional perspective of social identity, which posits that a group comes into existence when its members can identify with it and feel a sense of belonging [23]. Classical examples of this include belonging to a specific profession or previously belonging to a profession (such as in the case of veterans), age, gender, nationality or religious affiliation.

### 2.2 Diversity in entrepreneurship, entrepreneurial ecosystems and entrepreneurial education

Diversity can be defined as a collective measure of differences among members of a particular social grouping [24]. Such grouping may include a school class, a working group or a top-management team, while differences may refer to a person’s ethnic origin, age, gender, education, values or a combination of these factors [23]. The evolution of the concept and practice of diversity management can be traced back to affirmative action programs in the United States, which predominantly aimed to enhance career prospects and employment perspectives for individuals from ethnic minorities and women. However, contemporary diversity management has evolved to encompass a broader spectrum of dimensions beyond these two traditional facets [25]. The scope of diversity management extends beyond its business rationale for enhancing workforce productivity [26], and encompasses its role in facilitating isomorphic organizational change [27] and its capacity to formulate novel guidelines [28]. However, previous research on diversity management has not yet theorized the heterogeneity among identity categories, thus creating a research gap in terms of how multiple inequalities interact [29].

The development of the research field of diversity in entrepreneurship is contingent upon the identification and mitigation of significant gaps in extant literature. These gaps pertain to the definition and understanding of entrepreneurial diversity, as well as the paucity of reliable empirical studies [2]. Previous research underscores the necessity for research that embraces both diversity as well as complex typologies of entrepreneurship that transcend simplistic dichotomies, such as male vs female, entrepreneur vs. small business owner, opportunity vs. necessity-based, and similar [14]. Consequently, the concept of entrepreneurial diversity encompasses not only underrepresented groups within the entrepreneurial workforce but also the heterogeneity among existing entrepreneurs and the pursuit of comprehensive entrepreneurial typologies that span multiple dimensions [30]. Moreover, extant entrepreneurship literature has recognized that the utilization of multiple dimensions can provide a framework for both a granular analysis as well as a critical examination of the interconnections and interdependencies between social categories [12].

The extant literature on entrepreneurial diversity encompasses a wide range of variables but a has a paucity of meaningful and usable theoretical approaches [3]. One stream of previous diversity literature has identified four levels of diversity, which can serve as an analytical framework in the context of entrepreneurial diversity studies- namely the personality (the way of thinking and behaving), internal dimensions (age, gender, ethnicity, race), external dimensions (geographic location, income, educational background, parental status) and organisational dimensions (functional level, work content, seniority, work location) [3,31]. Conversely, another stream of previous diversity management literature has identified two relevant dimensions of diversity: observable attributes (surface level) attributes and underlying (deep level) attributes [5,6,32]. These two identified dimensions of diversity correspond well with the two out of four previously identified levels of diversity in entrepreneurship, namely the internal and external dimensions. In light of this extant research framework, the present study employs observable (age, gender) and underlying (previous school success and parental academic achievement) attributes of diversity that are pertinent in the context of entrepreneurial education.

### 2.3 Entrepreneurial intention and diversity in the university context

Entrepreneurial intention in the university context is a well-researched phenomenon in the previous entrepreneurship literature [33,34]. Proper design and execution of entrepreneurial education has been demonstrated to have a significant effect on the entrepreneurial intention of both male and female students [11,35]. However, the extant literature on female entrepreneurship is deficient in both a robust theoretical foundation and a rigorous methodological approach that can effectively address the tendency to ascribe underperformance to women relative to men and to position them as “lesser than” [12]. To address these shortcomings, new theoretical frameworks and methodological techniques are required. In this sense, recent research has identified that socio-cultural factors are the most important factors influencing the women entrepreneurs’ own perception of success [36]. These findings underscore the significance of social identity perspectives in gendered entrepreneurial research. In a similar vein, recent research grounded in social role theory has revealed that gender stereotypes influence entrepreneurial intentions of adults, contingent on their gender role orientation [37]. Moreover, studies have demonstrated that commercial, high-growth entrepreneurship is being predominantly perceived as male-like, while social entrepreneurship is perceived similar to both men and women [38].

First-generation students are defined as individuals whose parents do not have a college or university degree, and they are generally more likely to be women and tend to be older [39]. Research indicates that first-generation status at the university significantly reduces entrepreneurial intention [40,41], making it a relevant aspect of success in entrepreneurial programs in the university context. Previous literature offers explanations and research on this phenomenon through a cultural mismatch theory [42].

The extant literature on the relationship between prior academic performance and entrepreneurial intentions is inconclusive and characterized by conflicting findings. Prior research on entrepreneurship in an academic setting has indicated that low academic performance is a precursor to high entrepreneurial intention [43]. In contrast with these findings, previous research on the relationship between academic performance and entrepreneurial attitudes among K-12 students yielded no significant correlation between the two variables [44]. Additionally, previous research has identified that STEM entrepreneurs are more likely to hail from academically rigorous schools, while social entrepreneurs are more likely to come from schools with lower academic rigor and higher socioeconomic diversity [45].

## 3. Research framework

The goal of the research is to enhance the understanding on the role that the entrepreneurship support at the university has in supporting the entrepreneurial intention from a diversity and hence social identity perspective. The research framework is, therefore, based in the social identity theory. This theory provides a basis for the more complex interaction between entrepreneurial support by university and observable (gender, age) and non-observable diversity factors (previous school success, parent’s education attainment), as factors influencing entrepreneurial intention.

The research framework regarding diversity factors that relate to the entrepreneurial education and their impact on entrepreneurial intention is presented in the Table 1 below. The framework is based in the social identity theory. This theory posits that age and gender are the most salient predictors of group formation in the organisational contexts [15,16]. Gender, age, academic diversity and family household diversity have all been researched before in a classification of previous entrepreneurship research, but with a need for better theoretical foundation for research [3]. However, the focus of the present research was on determining the role of previous academic achievement and parents’ academic achievement on the formation of entrepreneurial intention. Higher academic achievement is expected to have a negative effect on entrepreneurial intention in line with the previous research [46].

**Table 1:** Diversity factors framework based in the social identity theory and classified in the diversity management literature.

Diversity factors of students in the entrepreneurial university used in this study	Attribute type- diversity management literature [5,6]	Social identity theory
Gender	Observable	Gender and age are considered to be strongest predictors of group formation in organisations, relevant both for identity theory and diversity management theory [15].
Age		
High-school grades (as an indication of academic aspirations)	Underlying (non-observable)	Academic achievement is a relevant category for researching antecedents of entrepreneurial intention in an entrepreneurial education context [46–48]. Parents academic achievement is a relevant category for researching antecedents of entrepreneurial intention in an entrepreneurial education context [49]. Entrepreneurial social identity is shaped by socialisation in terms of school peer grouping and parental influence [50].
Parents education level (indication of “first-generation status” at the university)		

The research is based in the social identity theory. However, having in mind the lack of research frameworks in the field of social identity theory in entrepreneurial education research, diversity management literature represents an important link between numerous possible antecedents or social identities of entrepreneurial intention and classifies them into two broad categories. The previous diversity management literature classifies diversity attributes into two broad categories, namely the observable diversity attributes (gender, age) and non-observable diversity attributes (socioeconomic and educational, occupational attributes) [5,6]

**Fig 1:**
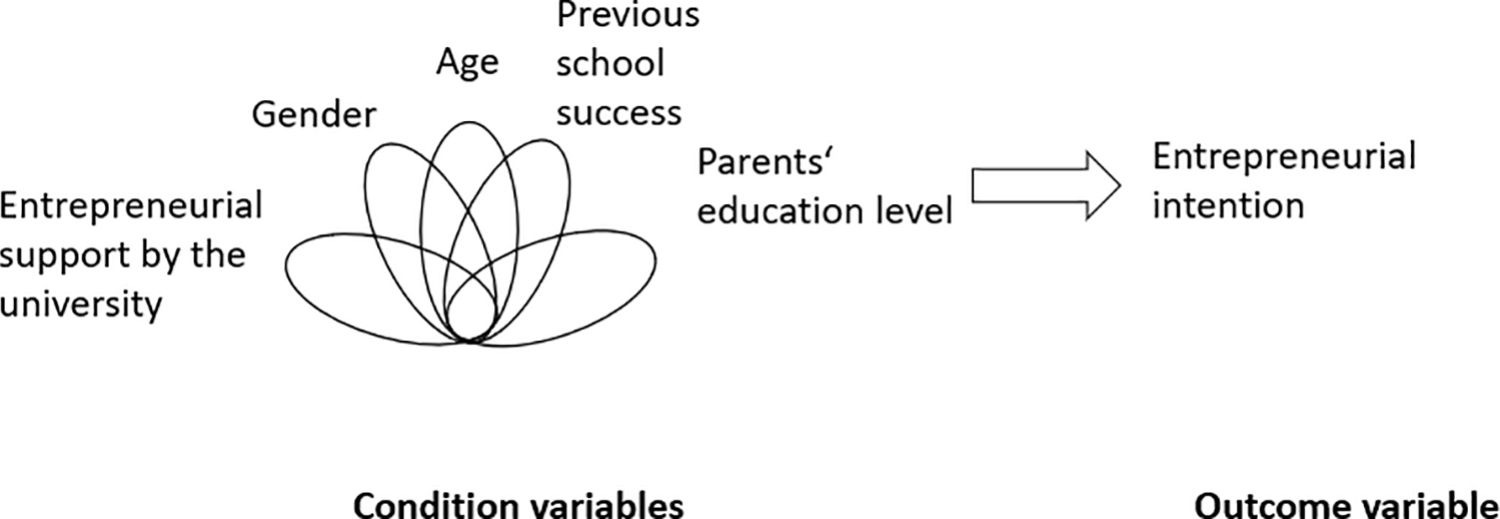
Research design of the study which includes a set of 5 conditions for diverse entrepreneurial education (based on social identity theory) and entrepreneurial intention as outcome variable.

The present research utilizes a configurational analysis to examine how (perceived) entrepreneurial support interacts with observable diversity factors (Gender, Age) and underlying diversity factors (Parents’ educational level and Previous school success-GPA). The research design is presented in the Fig 1 below.

## 4. Methodology

The present research uses the fsQCA methodology as a suitable method for researching entrepreneurial intention. Previous literature highlights the inherent complex causality of entrepreneurial intention [51]. In comparison to other potential approaches, such as correlational theorizing, which is predominant in the management research, the configurational approach is better suited to explaining complex causal phenomena and is well suited for advancing theoretical discourse in the domain of management research [52].

The fsQCA analysis is based on a dataset from an Internet survey, which was conducted between March and June 2021 on the sample of 846 university students in Croatia and Serbia. The data was collected in two private universities, the Libertas University in Zagreb, Croatia and the Singidunum University in Belgrade, Serbia. The two universities are comparable in terms of status and financing as both are privately funded, independent universities, with campuses in capital cities as well as other campuses throughout the country. Libertas has another campus in Dubrovnik, while Singidunum has additional campuses in Valjevo, Novi Sad and Niš. The questionnaire was conducted in Serbo-Croatian, the only difference being that the Croatian version was administered in Latin script and the Serbian version in Cyrilic script.

The data collection followed the standard ethics approval procedure at the Singidunum and Libertas Universities at the time of the data collection. At the Singidunum University, this meant obtaining the approval of the dean of the university, while at the Libertas University this meant obtaining a written approval from the head of study program. An implied informed consent was obtained from the students, so that the filling out of the questionnaire after reading the information about the study goals meant that they agree to participate in the study. The data collection was conducted in accordance with the Declaration of Helsinki from 1964. The data set is available for download at 10.5281/zenodo.13692956.

In order to conduct a thorough investigation into the abstract constructs of „entrepreneurial support by university” and “entrepreneurial intention”, batteries of questions needed to be developed. They are explained in detail in the Table 2 below. The Construct “Entrepreneurship support by university” comprises 5 items, while the construct “Entrepreneurial intention” comprises 6 items.

**Table 2:** Batteries of questions for Entrepreneurship support by university and Entrepreneurial intention.

Construct/Battery	Item/Question	Source
Entrepreneurship support by university (condition)	ES 1: My course and programs have increased my understanding of attitudes, values and motivations of entrepreneurs	Understanding of Attitudes, values and motivations of entrepreneurs [59–61]
	ES 2: My course and programs have increased my understanding of activities necessary for starting own business	Activities necessary for starting-up [62]
	ES 3: My course and programs have improved my practical managerial skills for starting own business	Practical managerial skills for starting up [63] and writing business plans [55]
	ES 4: My course and programs have increased my networking capability	Networking capability [64,65]
	ES 5: My course and programs have increased my capability for recognizing opportunities.	Opportunity recognition capability [66] and opportunities search [55]
Entrepreneurial intention (outcome)	EI 1: I am willing to do anything to become an entrepreneur.	The scale developed by Liñán & Chen [56] and widely used in the recent years, e.g.: [57] [58]
	EI 2: My professional goal is to become an entrepreneur.	
	EI 3: I will do anything in order to start and manage my own private company.	
	EI 4: I am decisive to create my own company in the future.	
	EI 5: I am thinking about starting a business-company very seriously.	
	EI 6: In the future I intend to start my own business.	

Entrepreneurship support at the university should help spur the entrepreneurial discovery process and help create partnerships across regional borders [53]. Entrepreneurial or entrepreneurship support inside third mission of university encompasses both entrepreneurial education as well as further offers, such are start-up councelling and consultation, networking, offices and start-up capital provision [54]. Entrepreneurship support at the university can include business opportunity search in terms of new research and new markets, business plan creation mentoring and assessment, as well as various training and financial support activities [55]. The business opportunity search is reflected in the ES 5 and business plan creation mentoring and assessment in the ES 3. Out of the three important activities of entrepreneurship support by universities, as suggested by Arroyo-Vazquez et al. [55] we haven’t included only the financial support, as this is still a missing link in the entrepreneurial support process in Croatia and Serbia, and is therefore not relevant. In addition, the model developed by Arroyo-Vazquez et al. [55], incorporates financial support as a component of business launch assistance and entrepreneurship support element. However, we have augmented the model by incorporating education, as reflected in the ES 2 and proposed by Shvedova & Maevskaya [54]; attitudes and awareness, as reflected in the ES 1 and suggested by Shvedova & Maevskaya [54]; and networking as reflected in the ES 4 and suggested by Shvedova & Maevskaya [54].

The entrepreneurial intention construct is based on a widely used six-item scale, developed by Liñán & Chen [56] and further used in other studies, for example by Kyriakopoulos et al. [57] and Cardella [58].

The following section delineates the calibration procedure for questionnaire data, a prerequisite for conducting fsQCA, as illustrated in Table 3 below. One obvious advantage of fsQCA and calibration is the possibility to use binary/categorical, Likert-scale, clickstreams and multimodal data successfully [67]. Notably, the calibration procedure is underutilized in the social sciences despite its extensive application in other scientific domains, such as physics [68]. However, the practice of calibration is often met with criticism from the perspective of quantitative scholarship, where uncalibrated measures are still used and preferred [69]. Similarly to the qualitative research, researcher needs prior theoretical knowledge of the examined variables in order to make decisions in different stages of the fsQCA analysis, one of them being calibration [70]. Reliable and informed calibration is an essential step towards high reliability and replicability of all fsQCA studies, both for quantitative and qualitative, interview data [71].

**Table 3:** Calibration of the conditions.

	*Maximum (0.95)*	*Median (0.5)*	*Minimum (0.05)*
*Entrepreneurial support university*	*4.00*	*3.00*	*2.00*
*Gender (directly recoded without calibrate function)*	*2 (female)*	*–*	*1 (male)*
*Age*	*38.75*	*22.00*	*19.00*
*GPA high school*	*5.00*	*4.00*	*3.00*
*Parent educational level*	*4.92*	*3.92*	*2.92*
*Entrepreneurial intention*	*4.92*	*3.92*	*2.92*

With the exception of the variable gender, all conditions were calibrated in the fsQCA software through the “calibrate” function, where minimum, median and maximum values are defined and the function returns the calibrated values. Following the recommendations in the previous literature [72], we first calibrated our 5-point Likert scale as 4,3 and 2, instead of calculating the 0.95, 05 and 0.05 data point for entrepreneurial support by university and for entrepreneurial intention. However, this approach returned suboptimal consistency values especially for low entrepreneurial intention, so we opted for calculating the 0.95, 05 and 0.05 data point in Excel (through “PERCENTILE” function) before proceeding to use the “calibrate” function in fsQCA, as suggested to be the standard solution by previous literature [72]. For gender, no calibration was calculated, as it is a binary variable. However, the values were recoded from 2 for female and 1 male to 1 for female and 0 for male.

## 5. Results

A total of 846 students participated in the survey, comprising 435 students from Libertas International University in Zagreb (51.4%) and 411 students from Singidunum University in Belgrade (48.6%). The Libertas University is a relatively small institution with an enrollment of approximately 3,000 students, while Singidunum University is a medium-sized university with an enrollment of around 9,000 students. The descriptive statistics regarding the sample characteristics are presented in Table 4 below. This table presents data on university affiliation, year of study, age, average GPA in high school, entrepreneurial intention, entrepreneurship support by university, and parents’ education level.

**Table 4:** Descriptive statistics regarding the major characteristics of the sample.

Variable	Values and distributions					
University affiliation	Libertas University		Singidunum University			
	51.4%		48,6%			
Gender	Male		Female			
	40,10%		59,90%			
Year of study	First	Second	Third	Fourth	Fifth	Sixth
	19,70%	38,20%	24,00%	12,20%	5,80%	0,10%
Age	<=20	<=30	<=40	<=50		
	22,34%	68,20%	5,32%	4,14%		
Average GPA in high school		=2	<=3	<=4	<=5	
		0,24%	13,71%	59,10	26,95	
Entrepreneurial intention	=1	<=2	<=3	<=4	<=5	
	2,13%	7,09%	21,99%	34,48%	34,04%	
Entrepreneurship support by university	=1	<=2	<=3	<=4	<=5	
	1,42%	4,73%	21,16%	43,26%	29,43%	
Parents‘ education level (average mother and father)	=1 Elementary school (8 years)	<=2 High school (3–4 years)	<=3 University or equivalent	<=4 Master and doctorate		
	1,89%	49,88%	41,02%	7,21%		

The reliability and validity of the two constructs, namely entrepreneurial intention and entrepreneurial support by university, were evaluated through the calculation of Cronbach's alpha for reliability and the Average Variance Extracted (AVE) for validity. The Cronbach alpha for the construct “university entrepreneurship support” is 0.92, which exceeds the 0.7 threshold for good reliability. Nevertheless, it exceeds the threshold value of 0.9, indicating that some of the five items may be superfluous. It is conceivable that the same level of reliability could be achieved with three or four items. The AVE is 0.703, which is above the threshold value of 0.5, indicating good validity. Cronbach's Alpha for the construct of entrepreneurial intention is 0.95, which is higher than 0.7, indicating good reliability. However, it is also above the threshold value of 0.9, suggesting that some of the six items may be redundant and that similar reliability could be achieved with three, four, or five items. AVE is 0.769, above the threshold value of 0.5, demonstrating good validity of the construct.

Only the values of more than 4 cases and above the threshold value of 0.8 have been included for coding the outcome variable before running the analysis. Algorithm used by fsQCA software was Quine-McCluskey. Setting the PRI threshold in the truth table, as recommended in the literature by Pappas and Woodside [72], to 0.5 returned error “ERROR(Quine-McCluskey): The 1 Matrix Contains All Configurations”, so we opted for PRI of 0.4 as a threshold value for high entrepreneurial intention and 0.3 for low entrepreneurial intention, in order to resolve the error code. The PRI of 0.4 (high entrepreneurial intention) and 0.3 (low entrepreneurial intention) led to higher frequency cut-off of 5. The presented solutions in the results are the intermediate solutions with core conditions from the parsimonious solution been highlighted with bigger circles. The smaller circles are additional conditions from the intermediate solution. ●/・- Core/contributory condition present; ◯/○- Core/contributory condition absent.

The first step in the analysing the single conditions that contribute to high and low entrepreneurial intention was to identify the conditions that are required for the outcome of interest to happen, as presented in the Table 5 below. These factors are named necessary conditions in fsQCA and imply a very strong relationship with the outcome variable. A generally recommended threshold value for consistency of necessary conditions is 0.9, as suggested by Ragin [73]. In this sense, only one condition fulfils this criterion: low parental educational level (0.90 consistency, 0.54 coverage) as a necessary condition for low entrepreneurial intention of students. Below the threshold value, but close and therefore noteworthy, is the high parental education level (0.87 consistency, 0.91 coverage) as a necessary condition for high entrepreneurial intention of students.

**Table 5:** The results of the necessity analysis for high/low entrepreneurial intention of university students at Libertas and Singidunum universities.

	High entrepreneurial intention		Low entrepreneurial intention	
	Consistency	Coverage	Consistency	Coverage
Gender (Female)	0.58	0.51	0.63	0.31
Gender (Male)	0.43	0.56	0.37	0.26
Age (old)	0.57	0.68	0.63	0.41
Age (young)	0.72	0.69	0.70	0.36
Entrepreneurial support university (high)	0.72	0.75	0.60	0.29
Entrepreneurial support university (low)	0.57	0.61	0.73	0.58
GPA high school (high)	0.70	0.66	0.71	0.37
GPA high school (low)	0.58	0.69	0.60	0.39
Parent educational level (high)	0.84	0.91	0.46	0.22
Parent educational level (low)	0.49	0.51	0.90	0.54

The results relating to the sufficiency analysis point to whether particular configurations are sufficient to lead to the observed outcome. Sufficiency analysis results are summarized and presented in a truth table in a Table 6 below, as a standard way of presenting the data. Considering that the through table deals with both presence (high) and absence (low) of an outcome variable and the fact that there are 5 researched conditions, there are 2^5^=32 possible combinations. After carefully inspecting the data, we decided to set the frequency cut-off at 4 and consistency cut-off at 0.8, but the exclusion of rows with low PRI values increased the frequency threshold to 5.

**Table 6:** Configurations sufficient for high/low entrepreneurial intention of university students at Libertas and Singidunum universities (truth table).

Condition variables	High entrepr. intention			Low entrepr. intention			
	Sol. 1	Sol. 2	Sol. 3	Sol. 1	Sol. 2	Sol. 3	Sol. 4
Entrepreneurial support by university		●	●	◯			
Gender (1-female, 0-male)			◯		●		●
Age		◯		◯		●	●
Previous school success (GPA)	・	○	○		○	○	
Parents’ education level	●	◯	◯	○	○	○	○
Consistency	0.89	0.85	0.85	0.88	0.83	0.85	0.91
Raw coverage	0.68	0.33	0.17	0.54	0.34	0.44	0.36
Unique coverage	0.41	0.03	0.01	0.13	0.02	0.05	0.06
Solution coverage	0.76			0.76			
Solution consistency	0.83			0.85			
Consistency cut-off	0.82			0.80			
Frequency cut-off	5			5			

The analysis revealed three distinct solutions (Sol. 1–3) associated with high entrepreneurial intention (Solution coverage 0.76, Solution consistency 0.83, Consistency cut-off 0.82 and Frequency cut-off 5). The most consistent configuration (Sol. 1, consistency = 0.89, raw coverage = 0.68) indicates that parents’ education level (PEL) is a core condition facilitating high entrepreneurial intention in combination with high previous school success (GPA) as a contributing factor. Solution 2 configuration includes entrepreneurial support by university (ES), young age (A) and low parents’ education level as core conditions coupled with low previous school success (GPA) as a contributing factor. Solution 3 points to a configuration where entrepreneurial support by university (ES), male gender (G) and low parents’ education level as core conditions are coupled with low previous school success (GPA) as a contributing factor.

Similarly, four distinct solutions were found to be sufficient for low entrepreneurial intention (Solution coverage 0.76, Solution consistency 0.85, Consistency cut-off 0.80 and Frequency cut-off 5). The Solution 1 is the most prevalent solution (raw coverage=0.54, consistency 0.88) and it highlights the combination of low entrepreneurial support by university (ES) and young age (A) as core conditions coupled with parents’ education level (PEL) as a contributing condition. Solution 2 points to a female gender (G) as a core condition with low previous school success (GPA) and low parents’ education level (PEL) as contributing conditions. Solution 3 depicts older age (A) as a core condition with low previous school success (GPA) and low parents’ education level as contributing conditions. Solution 4 is similar to solutions 2 and 4 and it points to the female gender (G) and older age (A) as core conditions with low parents’ educational level (PEL) as a contributing condition.

**Fig 2:**
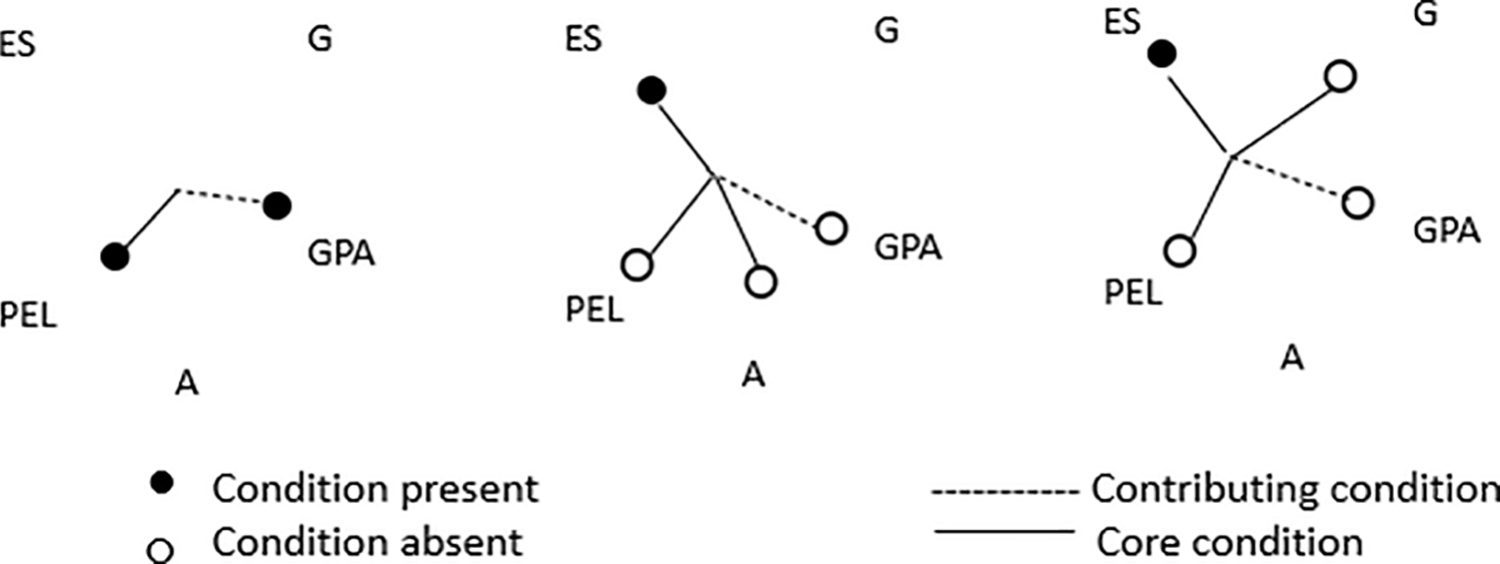
Configurations sufficient for high entrepreneurial intention of university students.

The results relating to high entrepreneurial intention (Fig 2 below) highlight the importance of high entrepreneurial support by university for students with lower parents‘ educational level (so-called first-generation students) who are either young or those of male gender. In both these cases, low previous school GPA plays a contributing role. For students from academic parental context and high previous school GPA a high entrepreneurial intention can be expected even without entrepreneurial support by university.

**Fig 3:**
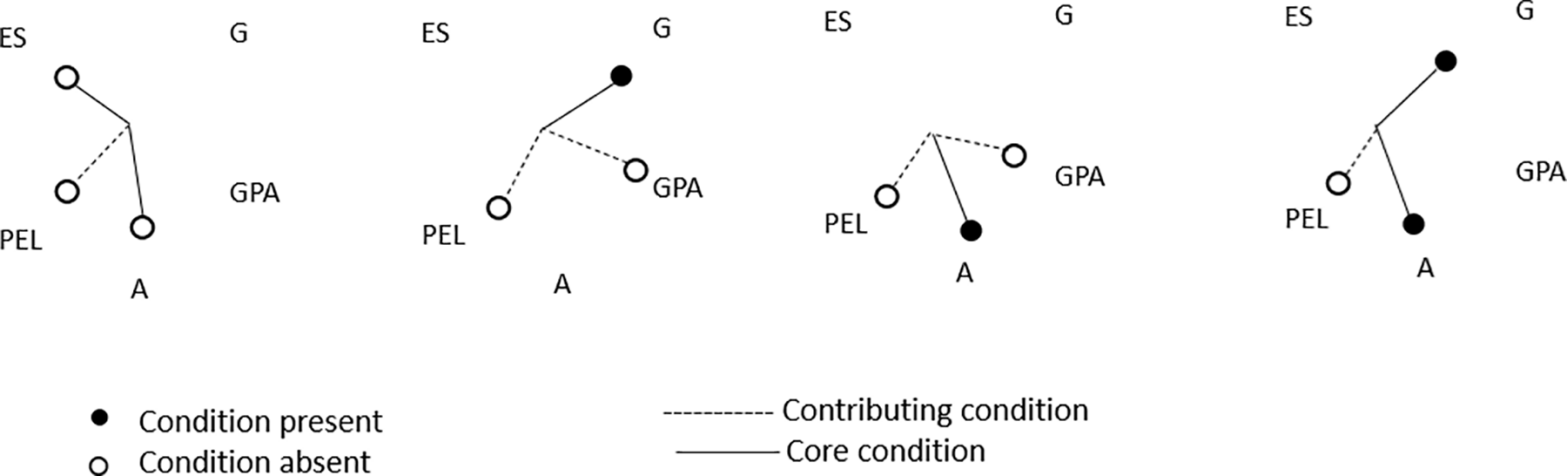
Configurations sufficient for low entrepreneurial intention of university students.

The results relating to low entrepreneurial intention (Fig 3 below) highlight the relevance of low entrepreneurial support by university, especially for younger students, where low parents’ education level plays a contributing role. There are also two solutions where female gender status is a core condition of low entrepreneurial intention, in one solution coupled with low previous school success and lower parents’ educational level and in another coupled with older age (A) as a core condition and low parent’s educational level as a contributing condition.

Overall, the results highlight the importance of entrepreneurial support by university of the creation of entrepreneurial intention especially for younger students and those from non-academic contexts. The lack of entrepreneurial support is also very relevant for low entrepreneurial intention in younger students from non-academic contexts. Entrepreneurial support is relevant for supporting male students in attaining entrepreneurial intention, while it has no role in low entrepreneurial intention of female students. Parents’ education level is the most consistent factor across all solutions and has a very important role in the creation of high entrepreneurial intention, even without entrepreneurial support by university. Low educational attainment of parents has a contributing role in the low entrepreneurial intentions of university students across solutions and is also a necessary condition for low entrepreneurial intention.

## 6. Discussion, limitations, and future research directions

The present study contributes to the field of research on entrepreneurial diversity by providing a social identity perspective to diversity in the formation of entrepreneurial intention in the university context. The present study addresses a significant research gap identified in the field of entrepreneurship research by Graham and Bonner [1], namely the lack of understanding of entrepreneurial diversity among untypical entrepreneurs, defined as individuals who lack high self-efficacy and/or entrepreneurial role models. This research gap has implications for both theoretical understanding and practical applications. To address this gap, the present study starts with social identity theory and diversity management framework and uses a configurational approach through fsQCA to examine the configurational paths leading to both high and low entrepreneurial intention among university students.

### Theoretical contributions/implications

The results of the present study could not directly confirm the assertion that entrepreneurial education is a crucial factor in fostering entrepreneurial intention of female students [8,9]. The results of the present study contribute to the existing research on this topic by confirming the importance of entrepreneurial education for first-generation male students and first-generation young students. There are two paths of low entrepreneurial intention of female students, where the lack of entrepreneurial support by university does not play a role, but rather a parents education level or a first-generation status appears to be a contributing factor to low entrepreneurial intention.

These results also confirm the relevance of using configurational approach through fsQCA in entrepreneurship diversity research and the research on interaction of social identities. The resulting configurations not only allow complex interpretations but also asymmetrical ones. This confirms the findings from the previous literature on the methodological foundations of fsQCA [74] and it’s suitability for entrepreneurship research [75]. Furthermore, they provide a means of discussing entrepreneurial diversity beyond dichotomies, closing a research gap identified in the previous entrepreneurship diversity research by Welter et al. [14].

The findings of the present study are highly pertinent to the reframing and further expansion of the classification efforts pertaining to diverse social identities in entrepreneurship. The provision of entrepreneurial support by a university represents a core condition in two distinct pathways. In the first pathway, this support is a core condition together with young age, low parents’ educational level and contributory condition of low previous school success. In the second pathway, the entrepreneurial support by university is a core condition together with male gender status, low parents’ education level and a contributory condition of low previous school GPA. This confirms the findings in the previous literature, where school teachers appear to see potential for entrepreneurship explicitly in those boys who are not academically accomplished but have other talents [48]. In this previous research, high-performing students and girls are not included in what school teachers define as “entrepreneurial type” of student. The results from the present study also confirm this finding, as our most consistent configuration for high entrepreneurial intention is the one where high parent’s educational level is a core condition and high previous school success a contributory condition, while university support plays no role in this path.

The results in the present study are inconclusive in terms of the role that previous school success has in the formation of entrepreneurial intention and therefore questions the finding of the previous research that low academic performance is an antecedent of high entrepreneurial intention [43]. These findings of the present study confirm the findings of the previous research that found no linear connection between the academic performance and entrepreneurial attitudes [44]. While the results of the previous study focused on Grade 12 high school students, the present study focuses on the impact of high-school GPA on entrepreneurial intention among student population. This paves the way for promising future research avenues with longitudinal designs on the evolution and advancement of entrepreneurial attitudes from high school to university and beyond, across diverse professional fields and education levels.

The absence of (or low) entrepreneurial intention, in the present research, demonstrated the lack of entrepreneurial support by university as a core condition for young students, where low parents’ education level is a contributory condition. Other two pathways include female gender as a core condition interacting with other contributory and core factors, which opens the question on the role of entrepreneurial career in female career planning. Although entrepreneurship support (or lack of it) are not included in these paths, it is notable that there is one configuration for high entrepreneurial intention where male gender is a core condition, while for low entrepreneurial intention there are two solutions where female gender is a core condition, one of them with older age as another core condition. These findings corroborate those of previous studies in the literature, which have highlighted the necessity for greater attention to be paid to diversity considerations in entrepreneurship education. This should encompass both the encouragement of older entrepreneurship, often referred to as “silver entrepreneurship,” and the promotion of female entrepreneurship. These results contribute to the previous findings of Ratten [41] regarding older entrepreneurship and Gerke et al. (2023) regarding female entrepreneurship.

### Practical implications

The present study has several important managerial implications for entrepreneurship education and entrepreneurial support provided by universities. The managerial implications are primarily related to the challenges of diversity management within entrepreneurial university tasks, both in terms of entrepreneurial education and beyond- including entrepreneurial incubators, accelerators and similar initiatives.

The findings of the present study indicate that diversity management aspects of entrepreneurial education and support are of outmost importance. A well-designed and implemented entrepreneurial support at a university should operationalize and track indicators of progress for diversity management measures. These indicators should go beyond the categories presented in this study to accommodate for the regional uniqueness. Each university and each entrepreneurial ecosystem face distinct challenges in terms of diversity management which should be reflected in the careful selection of the relevant measurement metrics.

### Limitations

It is important to note that the study is subject to several methodological limitations. The context of the study is situated within the broader regional context of the Western Balkans, with a particular focus on the case studies of Croatia and Serbia. The results should be interpreted with caution due to the geographical and linguistic limitations of the context where the two private universities are located. The fact that both universities are private institutions may impose limitations on the ability to make direct comparisons with public universities. Private universities are typically oriented towards teaching and community engagement (what is commonly referred to as the “first mission” and the “third mission,” respectively), whereas public universities are primarily research-intensive. As a result, generalizability of the findings to the broader higher education sector, particularly to public universities should be done with outmost caution. The selection of diversity factors is pertinent to the study area, comprising the two largest urban regions in the Western Balkans. However, their relevance may vary in contexts where other significant diversity factors are at play, such as racial, ethnic, linguistic, immigrant, or indigenous diversity.

### Future research directions

The present research demonstrated the relevance of the social identity-based approach to the formation of entrepreneurial intention and entrepreneurial education. Future research should build upon the social identity and (observable and underlying) diversity framework approach put forth in the present study. In this context of multiple diversity dimensions, entrepreneurial social identity and diversity also correspond well with “everyday entrepreneurship” identified in the previous research, in an attempt to move beyond binary classification of entrepreneurial success [14]. It is only by empirical validation and testing of the diverse set of conditions relevant to the entrepreneurial process that the theoretical conceptualization can be advanced: whether in the context of entrepreneurship, entrepreneurship education or the broader ecosystem of entrepreneurial support.

Future research in this field could adopt an experiential approach to entrepreneurial education, investigating the impact of different and innovative entrepreneurial support programs on the entrepreneurial intention of diverse groups of students or potential entrepreneurs. The research stream on innovative learning methods in entrepreneurship education represents a novel and promising area of inquiry with considerable potential for further exploration [76]. Furthermore, it lends itself well to integration with an entrepreneurial diversity perspective, which could facilitate more precise measurement of the impact of these educational programs on entrepreneurial intention.

Another promising extention of the present approach would be the inclusion of parents’ entrepreneurial influence, as it could be a critical link in forming student entrepreneurial identity. Previous literature determined that having self-employed parents, and generally parental entrepreneurial activity was associated with higher entrepreneurial intention, but not with higher entrepreneurial alertness [51,77].

## 7. Conclusions

The present study is predicated on the social identity theory, and its objective is to examine the interaction of observable and underlying diversity factors in the process of entrepreneurial support at the university in the formation of student entrepreneurial intention. Therefore, the results of the present study are of relevance both for social identity literature in entrepreneurship, as well as entrepreneurial diversity research.

The results of the study showcase the multidimensionality of social identities and their interactions with entrepreneurial support of university in the process of entrepreneurial intention formation. The findings of the study indicate that low parents’ education level and therefore first-generation status at the university was identified as a necessary condition for low entrepreneurial intention of university students. Furthermore, the study underscores the pivotal role of entrepreneurial support by university in the creation of entrepreneurial intention for younger students and those with first-generation status. Similarly, the lack of entrepreneurial support is also very relevant for low entrepreneurial intention in younger students with first-generation status. The research results contribute to better understanding of the interaction of social identities or observable and underlying diversity factors in entrepreneurship in a university context.
